# Clinical characteristics and prognostic factors of ovarian granulosa cell tumors: a retrospective cohort study

**DOI:** 10.3389/fonc.2025.1640232

**Published:** 2026-01-14

**Authors:** Yinbai Fan, Yanzhen Zeng

**Affiliations:** 1 Department of Physical Examination, Ganzhou People’s Hospital, Ganzhou, Jiangxi, China; 2 Department of Obstetrics, Ganzhou People’s Hospital, Ganzhou, Jiangxi, China

**Keywords:** fertility-sparing surgery, FIGO staging, ovarian granulosa cell tumor, prognostic factors, relapse-free survival, sex cord-stromal tumor

## Abstract

**Background:**

Ovarian granulosa cell tumors (OGCTs) are rare sex cord-stromal tumors comprising distinct histopathological entities: adult-type and juvenile-type GCTs. Comparative data on clinical characteristics and outcomes between these subtypes remain limited.

**Methods:**

A retrospective analysis was performed on 29 patients diagnosed with OGCTs (26 adult-type and 3 juvenile-type) from January 2008 to December 2024. Clinical data, tumor markers, pathological features, surgical approaches, and survival outcomes were assessed. Relapse-free survival (RFS) was evaluated using Kaplan-Meier analysis. Given the small juvenile cohort (n=3) with zero recurrence events during follow-up and distinct biological characteristics, prognostic factor analysis using Cox regression was restricted to adult-type GCTs (n=26, 3 recurrence events).

**Results:**

OGCTs exhibited distinct age-related patterns, with adult-type tumors primarily affecting postmenopausal women (median age 50.5 years) and juvenile-type tumors occurring in pediatric patients (median age 13.0 years) (p = 0.005). Most patients (89.7%) presented with FIGO stage I disease, with stage IC being the most common (55.2%). Abdominal mass was the most frequent presenting symptom (51.7%). Immunohistochemically, both subtypes showed high positivity for inhibin-α (93.1%), calretinin (92.3%), and CD99 (95.0%). Fertility-sparing surgery was performed in all juvenile-type cases (100%) compared to 19.2% of adult-type cases (p = 0.018). Recurrence occurred in three patients (10.3%), all of whom had adult-type tumors. In adult-type GCTs, advanced FIGO stage (IC-III) was the only independent prognostic factor for RFS in multivariate analysis (HR 13.57, 95% CI 1.19-154.3, P = 0.035). Preoperative CA125 showed a trend (HR 1.04 per U/mL, P = 0.079) that did not reach statistical significance.

**Conclusions:**

Within the constraints of limited follow-up (median 60 months) and small sample size, early-stage OGCTs demonstrate favorable short- to intermediate-term outcomes, with FIGO stage as the primary prognostic determinant in adult-type disease. Fertility-sparing surgery appears feasible in early-stage disease, particularly for juvenile-type tumors. However, the short follow-up duration likely underestimates true long-term recurrence rates in adult-type GCTs, which characteristically recur late.

**Limitations:**

Small sample size (particularly juvenile cohort, n=3), single-center design, relatively short follow-up (median 60 months; 72% <8 years), and absence of GCT-specific tumor markers (Inhibin B, AMH) limit generalizability and assessment of long-term outcomes.

## Introduction

Ovarian granulosa cell tumors (OGCTs) are rare sex cord-stromal tumors, accounting for approximately 2-5% of all ovarian malignancies (1, 2). These tumors are known for their ability to produce estrogen and their potential for late recurrence, necessitating long-term follow-up for effective patient management (3). GCTs are classified into two distinct histological subtypes: adult-type and juvenile-type, which differ notably in clinical presentation, biological behavior, and prognosis (4).

Adult-type GCTs typically affect postmenopausal women and exhibit indolent growth patterns, with the possibility of recurrence occurring many years, or even decades, after the initial diagnosis (5, 6). In contrast, juvenile-type GCTs predominantly occur in children and young adults, often presenting with precocious puberty in prepubertal patients, and may demonstrate more aggressive biological behavior in certain cases (7).

The rarity of granulosa cell tumors (GCT) has limited comprehensive studies on their clinical characteristics and prognostic factors (8). Current treatment approaches are largely based on small case series and expert consensus rather than large-scale, evidence-based research (9, 10). Surgical resection remains the cornerstone of treatment, with fertility-sparing surgery being a significant consideration for younger patients (11, 12). However, the optimal extent of surgical intervention and the role of adjuvant chemotherapy remain areas of debate (13).

Several factors have been suggested as potential prognostic indicators for GCTs, including patient age, tumor stage as per the International Federation of Gynecology and Obstetrics (FIGO) staging system, tumor size, and various serum biomarkers (14). Tumor markers such as CA125, HE4, and the risk of ovarian malignancy algorithm (ROMA) have shown promise in other ovarian malignancies, but their utility in GCTs warrants further investigation (15, 16).

In light of the limited comprehensive data on the clinical features and prognostic factors of ovarian GCTs, this retrospective cohort study analyzed the clinicopathological features, treatment outcomes, and survival patterns of patients with these tumors. This study aims to compare the clinical characteristics of adult-type and juvenile-type GCTs and identify independent prognostic factors associated with relapse-free survival.

## Methods

### Study design and patient selection

This retrospective cohort analysis was conducted at Ganzhou People’s Hospital between 2008 and 2024. The study protocol was approved by the institutional ethics committee, which waived the requirement for informed consent due to the retrospective nature of the study and the use of fully anonymized clinical data. Patients diagnosed with OGCTs during this period were initially identified through the hospital’s electronic pathology database (n = 38).

**Figure 1:**
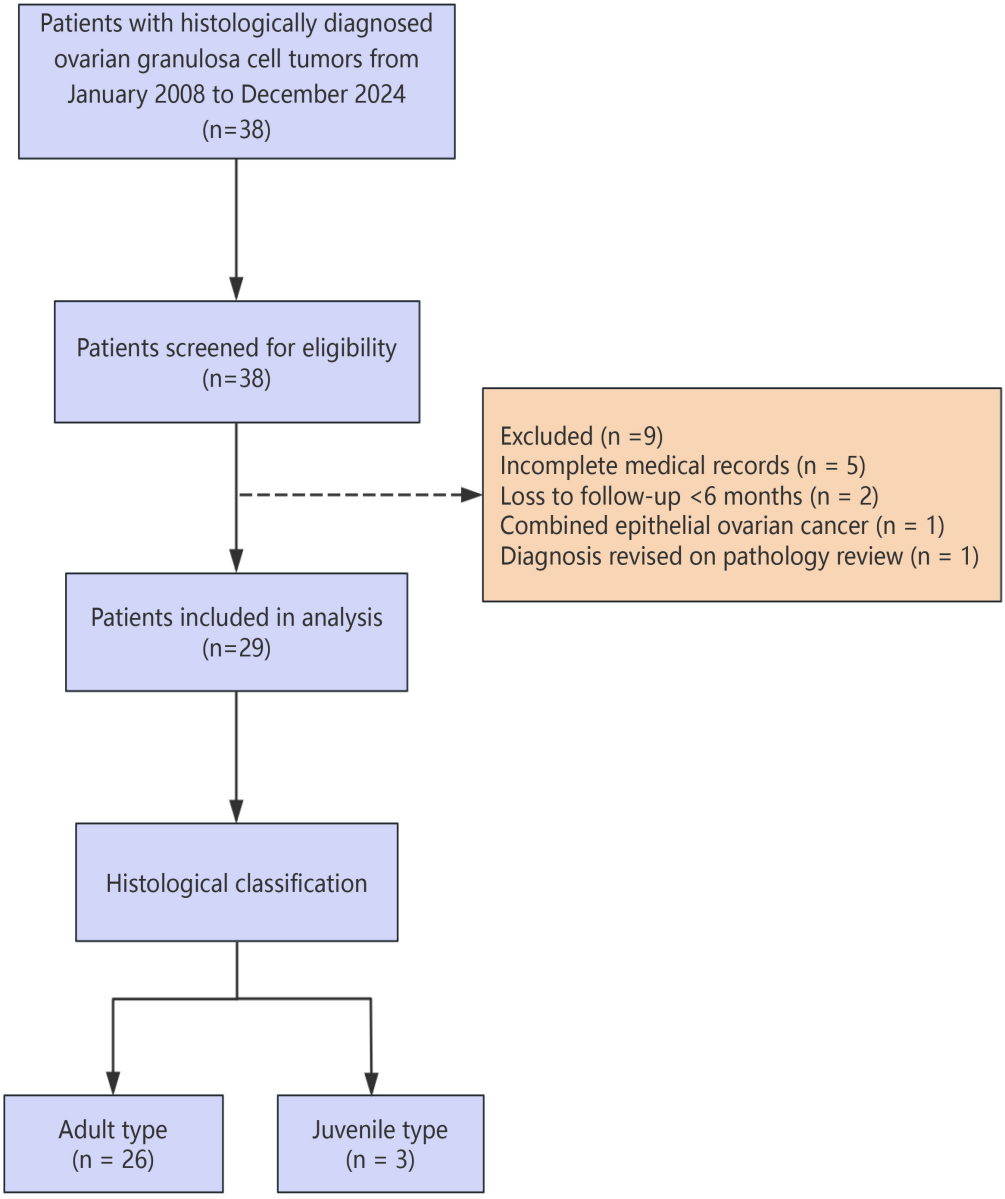
Flow chart of sample selection.

The patient selection flow is depicted in **Figure 1**. All 38 cases were screened for eligibility. Inclusion criteria were: (1) histologically confirmed OGCT; (2) complete medical records, including surgical details and pathological reports; (3) a minimum follow-up duration of 6 months or documented event. Exclusion criteria led to the exclusion of 9 patients: incomplete medical records (n = 5), loss to follow-up < 6 months without documented event (n = 2), concurrent epithelial ovarian cancer (n = 1), and diagnosis revised on pathology re-review (n = 1).

A total of 29 patients meeting all inclusion criteria were included in the final analysis (**Figure 1**). Selection was based solely on data availability and diagnostic confirmation, rather than clinical characteristics or outcomes, thus minimizing selection bias.

### Data collection

Clinical data were extracted from electronic medical records and included patient demographics, presenting symptoms, preoperative imaging findings, surgical details, pathological characteristics, adjuvant treatment, surgical staging, and follow-up information. Laboratory data, including complete blood count and tumor markers (CA125, HE4, AFP, CEA), were recorded when available. The calculated ROMA indices were documented. Inhibin A or inhibin B despite being a more specific marker for granulosa cell tumors, was not routinely tested during the study period and was available for only 6 patients (20.7%). Anti-Müllerian hormone (AMH), another GCT-specific marker, was similarly not routinely measured.

### Histopathological analysis

All cases were reviewed by experienced gynecological pathologists to confirm the diagnosis and classify tumors according to the World Health Organization criteria. Tumors were classified as either adult-type or juvenile-type based on established histological criteria (17).

Immunohistochemical staining was performed using standard protocols for markers, including calretinin, inhibin-α, cytokeratin (CK), vimentin (Vim), CD56, CD99, S-100, Ki-67, WT1, epithelial membrane antigen (EMA), and CA125.

### Staging and treatment

Tumors were staged according to the FIGO staging system. Surgical procedures were categorized as fertility-sparing surgery (unilateral salpingo-oophorectomy with or without omentectomy) or radical surgery (hysterectomy with bilateral salpingo-oophorectomy with or without staging procedures). Complete surgical staging for GCTs included a thorough inspection of the abdominal cavity, peritoneal cytologic washings, careful examination and biopsy of any suspicious peritoneal surfaces, and omentectomy when indicated. Given the extremely low incidence of lymph node metastases in GCTs, lymphadenectomy was not considered essential for complete staging, in accordance with current evidence and expert recommendations (18). Staging was considered incomplete if peritoneal assessment, cytologic washings, or inspection of the contralateral ovary were omitted.

The decision to administer adjuvant chemotherapy was based on the following criteria: FIGO stage II-III disease, tumor rupture during surgery, high mitotic index (> 10 mitoses/10 HPF), or incomplete surgical staging. Chemotherapy regimens included bleomycin, etoposide, and cisplatin (BEP); paclitaxel plus carboplatin (TC); etoposide plus cisplatin (EP); and paclitaxel plus cisplatin (TE), selected based on patient factors and institutional protocols.

### Follow-up and outcome assessment

Patients were followed regularly with clinical examination, imaging studies, and tumor marker assessments. Relapse-free survival (RFS) was defined as the time from the date of initial surgery to the first occurrence of any of the following events:

(1) Radiologically or histologically confirmed disease recurrence (local, regional, or distant), (2) death from any cause, or (3) last follow-up visit for patients remaining alive and disease-free. Recurrence was defined as radiological evidence of disease recurrence confirmed by imaging or histopathology.

### Statistical analysis

Descriptive statistics were presented as means ± standard deviations or medians with interquartile ranges for quantitative variables, and as counts with percentages for qualitative variables, depending on the distribution characteristics. Comparisons between adult-type and juvenile-type GCTs were performed using Student’s t-test or Mann-Whitney U test for continuous variables and Fisher’s exact test or Chi-square test for categorical variables, as appropriate. Given the small juvenile cohort (n=3) and distinct biological characteristics of the two subtypes, these comparisons were presented for descriptive purposes only, with appropriate caveats regarding statistical interpretation.

### Survival and prognostic factor analysis

Survival curves were constructed using the Kaplan-Meier method, and differences between groups were compared using the log-rank test. Given the small sample size of juvenile-type GCTs (n=3), zero recurrence events during the follow-up period (median 96 months, range 74–124 months), and the distinct biological characteristics of these entities, prognostic factor identification was restricted to adult-type GCTs (n=26, 3 recurrence events). Univariate Cox proportional hazards regression was performed to evaluate potential prognostic factors including age (<45 vs. ≥45 years), FIGO stage (I vs. II-III), tumor size (≤10 vs. >10 cm), completeness of surgical staging, adjuvant chemotherapy, and preoperative tumor markers (CA125, HE4, CEA, AFP, ROMA index). Variables with P<0.20 in univariate analysis were included in multivariate Cox regression models to identify independent prognostic factors. Hazard ratios (HRs) with 95% confidence intervals (CIs) were calculated to estimate risk.

For juvenile-type cases, outcomes were reported descriptively only due to insufficient sample size and absence of events during the observation period.

Statistical significance was defined as p < 0.05. All analyses were conducted using the R statistical platform (http://www.R-project.org, The R Foundation).

## Results

### Patient characteristics

This study included 29 individuals diagnosed with OGCTs, consisting of 26 adult-type (89.7%) and 3 juvenile-type (10.3%) tumors. The median age significantly differed between the two groups: 50.5 years (IQR: 47.0-61.0) for adult-type and 13.0 years (IQR: 10.0-14.5) for juvenile-type tumors. All three juvenile-type cases (100%) occurred in patients younger than 30 years, whereas the majority of adult-type cases (92.3%, 24/26) were diagnosed in patients aged 30 years or older. Specifically, among adult-type GCTs, 7.7% (2/26) occurred in patients <30 years, 42.3% (11/26) in those aged 30–50 years, and 50.0% (13/26) in patients >50 years.

The most common presenting symptom was abdominal mass (51.7%), followed by abdominal pain or bloating (20.7%) and vaginal bleeding (17.2%). There were no significant differences in presenting symptoms between adult-type and juvenile-type tumors (p = 0.36). The majority of patients (89.7%) presented with FIGO stage I disease, with no significant differences in stage distribution between the two histological subtypes (p = 1.0) (**Table 1**).

**Table 1:** Comparison of clinical characteristics between adult-type and juvenile-type ovarian granulosa cell tumors.

Variables	Total (n = 29)	Adult (n = 26)	Juvenile (n = 3)	P value‡
Age,years	49.0 (38.0, 58.0)	50.5 (47.0, 61.0)	13.0 (10.0, 14.5)	0.005
Age at diagnosis,years				0.001
< 30	5 (17.2)	2 (7.7)	3 (100)	
30 - 50	11 (37.9)	11 (42.3)	0 (0)	
> 50	13 (44.8)	13 (50)	0 (0)	
Initial Symptoms,				0.36
Abdominal mass	15 (51.7)	14 (53.8)	1 (33.3)	
Abdominal pain/bloating	6 (20.7)	5 (19.2)	1 (33.3)	
Vaginal bleeding	5 (17.2)	5 (19.2)	0 (0)	
Abdominal pain and mass	3 (10.3)	2 (7.7)	1 (33.3)	
FIGO Stage				1
I	26 (89.7)	23 (88.5)	3 (100)	
II-III	3 (10.3)	3 (11.5)	0 (0)	
CA125 elevation, n (%)				0.442
No	12 (41.4)	11 (42.3)	1 (33.3)	
Yes	14 (48.3)	13 (50)	1 (33.3)	
NA	3 (10.3)	2 (7.7)	1 (33.3)	
HE4 elevation, n (%)				0.335
No	17 (58.6)	16 (61.5)	1 (33.3)	
Yes	9 (31.0)	8 (30.8)	1 (33.3)	
NA	3 (10.3)	2 (7.7)	1 (33.3)	
Serum Markers				
RBC, ×10^9^/L	4.4 ± 0.7	4.4 ± 0.7	4.6 ± 0.2	0.612
Hemoglobin, g/L	122.5 ± 22.0	124.4 ± 20.5	106.3 ± 32.4	0.183
AFP, ng/mL	2.5 (1.6, 3.1)	2.9 (1.8, 3.5)	1.2 (1.2, 1.2)	0.089
CEA, ng/mL	2.0 (0.9, 2.6)	2.1 (1.2, 2.9)	0.8 (0.7, 0.8)	0.089
CD125, U/mL	33.7 (16.0, 85.7)	33.7 (14.5, 83.0)	77.7 (52.6, 102.7)	0.441
HE4, pmol/L	63.4 (45.4, 83.8)	54.1 (44.2, 80.8)	578.3 (578.3, 578.3)	0.097
Inhibin-a*, pg/mL	89.5 (39.6, 219.2)	98.7 (50.5, 234.1)	2.4 (2.4, 2.4)	0.104
ROMA1	11.4 (5.9, 23.0)	9.9 (5.6, 22.5)	96.9 (96.9, 96.9)	0.097
ROMA2	24.6 (10.2, 40.7)	22.6 (9.7, 39.1)	88.5 (88.5, 88.5)	0.097

FIGO, International Federation of Gynecology and Obstetrics; HE4, Human epididymis protein 4;ROMA1, Premenopausal ROMA index;ROMA2, Post-menopausal ROMA index

*Serum inhibin-a was not routinely measured; only 6 patients had available data.

‡Statistical comparisons should be interpreted cautiously due to small sample size in juvenile cohort (n=3). These subtypes represent distinct histopathological entities; comparisons are presented for descriptive purposes and hypothesis generation.

### Laboratory and tumor marker findings

CA125 elevation (> 35 U/mL) was observed in 48.3% of patients, with a median value of 33.7 U/mL (IQR: 16.0-85.7). Among patients with elevated CA125, the mean level was 95.15 U/mL. HE4 elevation was seen in 31.0% of cases. While no statistically significant differences in tumor marker levels were found between adult-type and juvenile-type tumors, juvenile-type tumors showed numerically higher median values for HE4 (578.3 vs. 54.1 pmol/L, p = 0.097) and ROMA indices.

Mean hemoglobin levels were 122.5 ± 22.0 g/L, with no significant difference between the groups. AFP and CEA levels were generally within normal ranges, with median values of 2.5 ng/mL and 2.0 ng/mL, respectively (**Table 1**).

### Pathological features and immunohistochemistry

Tumor size analysis revealed that 51.7% of tumors measured 5–10 cm, 34.5% were > 10 cm, and 13.8% were < 5 cm. All juvenile-type tumors (100%) measured 5–10 cm, while adult-type tumors exhibited a more varied size distribution (p = 0.337).

Immunohistochemical analysis showed high positivity rates for typical GCT markers: inhibin-α (93.1%), calretinin (92.3%), CD99 (95.0%), and CD56 (94.7%). Ki-67 proliferation index > 30% was observed in 14.8% of cases, all of which were adult-type tumors. No significant differences in immunohistochemical marker expression were found between adult-type and juvenile-type tumors (**Table 2**).

**Table 2:** Pathological features and treatment between adult-type and juvenile-type ovarian granulosa cell tumors.

Feature	Event (n)	Adult-Type (n=26)	Juvenile-Type (n=3)	P value
Tumor Size				0.337
<5 cm	4 (13.8)	4 (15.4)	0 (0)	
5–10 cm	15 (51.7)	12 (46.2)	3 (100)	
>10 cm	10 (34.5)	10 (38.5)	0 (0)	
Immunohistochemistry				
Calretinin (+)	24 (92.3)	22 (91.7)	2 (100)	1
Inhibin-a (+)	27 (93.1)	24 (92.3)	3 (100)	1
CK (+)	9 (34.6)	9 (37.5)	0 (0)	0.529
Vim (+)	21 (87.5)	19 (86.4)	2 (100)	1
CD56 (+)	18 (94.7)	17 (94.4)	1 (100)	1
CD99 (+)	19 (95.0)	16 (94.1)	3 (100)	1
s-100 (+)	7 (77.8)	5 (71.4)	2 (100)	1
Ki-67 index >30%	4 (14.8)	4 (16.7)	0 (0)	1
WT1(+)	12 (85.7)	11 (84.6)	1 (100)	1
EMA (-)	20 (100.0)	18 (100)	2 (100)	1
CA125 (+)	3 (75.0)	2 (66.7)	1 (100)	1
Surgery				0.018
Fertility-sparing surgery	8 (27.6)	5 (19.2)	3 (100)	
Hysterectomy + BSO	20 (69.0)	20 (76.9)	0 (0)	
Hysterectomy + BSO + staging	1 (3.4)	1 (3.8)	0 (0)	
Adjuvant Chemotherapy				0.024
Yes	12 (41.4)	12 (46.2)	0 (0)	
No	17 (58.6)	14 (53.8)	3 (100)	
Chemotherapy Regimen (n=12)				1
BEP	3 (25.0)	3 (25.0)	0	
TC	6 (50.0)	6 (50.0)	0	
EP	2 (16.7)	2 (16.7)	0	
TE	1 (8.3)	1 (8.3)	0	
Surgical Staging*				0.573
Incomplete	13 (44.8)	11 (42.3)	2 (66.7)	
Complete	16 (55.2)	15 (57.7)	1 (33.3)	
Median follow-up time (months)	60.0 (24.0, 96.0)	52.5 (19.5, 82.5)	96.0 (85.0, 110.0)	0.099

BSO, bilateral salpingo-oophorectomy; BEP, bleomycin, etoposide, cisplatin; TC, paclitaxel, carboplatin; EP, etoposide, cisplatin; TE, paclitaxel, cisplatin;

Among the 12 patients who received chemotherapy: 3 had stage II-III disease (all received chemotherapy), and 9 had stage I disease with high-risk features (tumor rupture, high Ki-67 index >30%, or incomplete surgical staging). *Complete staging defined as comprehensive peritoneal assessment with cytology, without requirement for lymphadenectomy given the low incidence of nodal metastases in granulosa cell tumors.

Beyond the expected age differences, the data revealed distinct clinical patterns: juvenile-type tumors uniformly presented with intermediate size (5–10 cm), were all treated with fertility-sparing surgery, and had longer disease-free intervals (median follow-up 96 months without recurrence). Adult-type tumors exhibited a more heterogeneous size distribution (ranging from < 5 cm to > 10 cm), predominantly underwent radical surgery (76.9%), and showed variable Ki-67 proliferation indices, with 16.7% exhibiting high proliferation (> 30%).

### Treatment and surgical management

Surgical treatment differed significantly between the two groups (p = 0.018). All patients with juvenile-type tumors (100%) underwent fertility-sparing surgery, while only 19.2% of adult-type patients received fertility-sparing procedures. The majority of adult-type patients (76.9%) underwent hysterectomy with bilateral salpingo-oophorectomy. One patient (3.4%) received comprehensive staging surgery, which included hysterectomy, adnexectomy, and staging procedures.

According to staging criteria appropriate for GCTs (excluding lymphadenectomy due to the low metastatic risk), complete surgical staging was performed in 16 patients (55.2%), while 13 patients (44.8%) had incomplete staging. Among adult-type tumors, 11/26 (42.3%) had incomplete staging, and 15/26 (57.7%) had complete staging. Among juvenile-type tumors, 2/3 (66.7%) had incomplete staging, and 1/3 (33.3%) had complete staging (p = 0.573) (**Table 2**).

In terms of adjuvant chemotherapy, 12 patients (41.4%) received postoperative chemotherapy, all of whom had adult-type tumors. The chemotherapy regimens included bleomycin, etoposide, and cisplatin (BEP regimen, n = 3), paclitaxel plus carboplatin (TC regimen, n = 6), etoposide plus cisplatin (EP regimen, n = 2), and paclitaxel plus cisplatin (TE regimen, n = 1). The decision for adjuvant chemotherapy was based on the following criteria: FIGO stage II-III disease, tumor rupture during surgery, high mitotic index, or incomplete surgical staging. All 3 patients with stage II-III disease received adjuvant chemotherapy, while 9 stage I patients received chemotherapy due to high-risk features, including tumor rupture or high Ki-67 index (> 30%). No juvenile-type patients received adjuvant chemotherapy, as all presented with stage I disease and underwent complete surgical resection (**Table 2**).

### FIGO stage distribution and clinical characteristics

The detailed FIGO stage distribution is presented in **Table 3**. The majority of patients (n = 26, 89.7%) presented with stage I disease at initial diagnosis. Among stage I patients, stage IC was the most common presentation, accounting for 16 patients (55.2% of the total cohort and 61.5% of stage I patients), followed by stage IB (n = 6, 20.7% of the total cohort) and stage IA (n = 4, 13.8% of the total cohort). Three patients (10.3%) presented with advanced-stage disease: one with stage IIA (3.4%), one with stage IIB (3.4%), and one with stage IIIC (3.4%). Notably, as shown in **Table 3**, all advanced-stage cases (stage II-III) occurred exclusively in the adult-type group (3/26 adult-type patients, 11.5%), while all three juvenile-type patients presented with stage IC disease.

**Table 3:** FIGO stage distribution and clinical characteristics.

FIGO Stage	N (%)	Adult-type	Juvenile-type	Recurrences
Stage I	26 (89.7%)	23	3	1
IA	4 (13.8%)	4	0	0
IB	6 (20.7%)	6	0	0
IC	16 (55.2%)	13	3	1
Stage II	2 (6.9%)	1	0	0
II A	1 (3.4%)	1	0	1
II B	1(3.4%)	1	0	0
Stage III	1(3.4%)	1	0	0
III C	1(3.4%)	1	0	1

Among adult-type tumors, 23 of 26 patients (88.5%) presented with stage I disease (stage IA: 4, stage IB: 6, stage IC: 13), while 3 patients (11.5%) had stage II-III disease (stage IIA: 1, stage IIB: 1, stage IIIC: 1). In contrast, all juvenile-type tumors (3/3, 100%) were diagnosed at stage IC. The concentration of advanced-stage disease exclusively in the adult-type group has significant implications for prognosis and treatment planning.

### Follow-up and survival analysis

Median follow-up duration was 60 months (range 9–168 months) for the entire cohort. Importantly, 75% of patients (21/29) had follow-up duration shorter than 8 years (96 months), and only 25% (8/29) were followed for 8 years or longer. Among the adult-type cohort specifically, median follow-up was 52.5 months (range 9–168 months), with 19 patients (73%) followed for less than 8 years. For the juvenile-type cohort, median follow-up was 96 months (range 74–124 months), with two patients (75%) having follow-up longer than 7 years (84 months). The maximum follow-up in this subgroup was only 10 years, which is insufficient to assess long-term outcomes even in juvenile-type GCTs.

During the follow-up period, 3 patients (10.3%) experienced disease recurrence, all with adult-type tumors. Details of the recurrences are provided in **Table 4**. The mean time to recurrence was 63.3 months (range: 10–102 months). All recurrences occurred in the pelvis. Of the 3 recurrence cases: Case 1 (stage IC, adult-type) had complete staging and recurred at 78 months; Case 2 (stage IIIC, adult-type) had complete staging and recurred at 10 months; Case 3 (stage IIA, adult-type) had incomplete staging and recurred at 102 months. Two of the 3 recurrence cases (66.7%) had complete surgical staging. No recurrences were observed in the juvenile-type group.

**Table 4:** Recurrence patterns in ovarian granulosa cell tumors.

Case	Age	Type	FIGO Stage	Surgical Staging	Adjuvant Chemotherapy	Time to Recurrence (months)	Site of Recurrence	Initial Inhibin
1	38	adult	IC	complete	no	78	Pelvis	Elevated
2	71	adult	IIIC	complete	yes	10	Pelvis	Elevated
3	49	adult	IIA	incomplete	yes	102	Pelvis	Unknown

**Figure 2:**
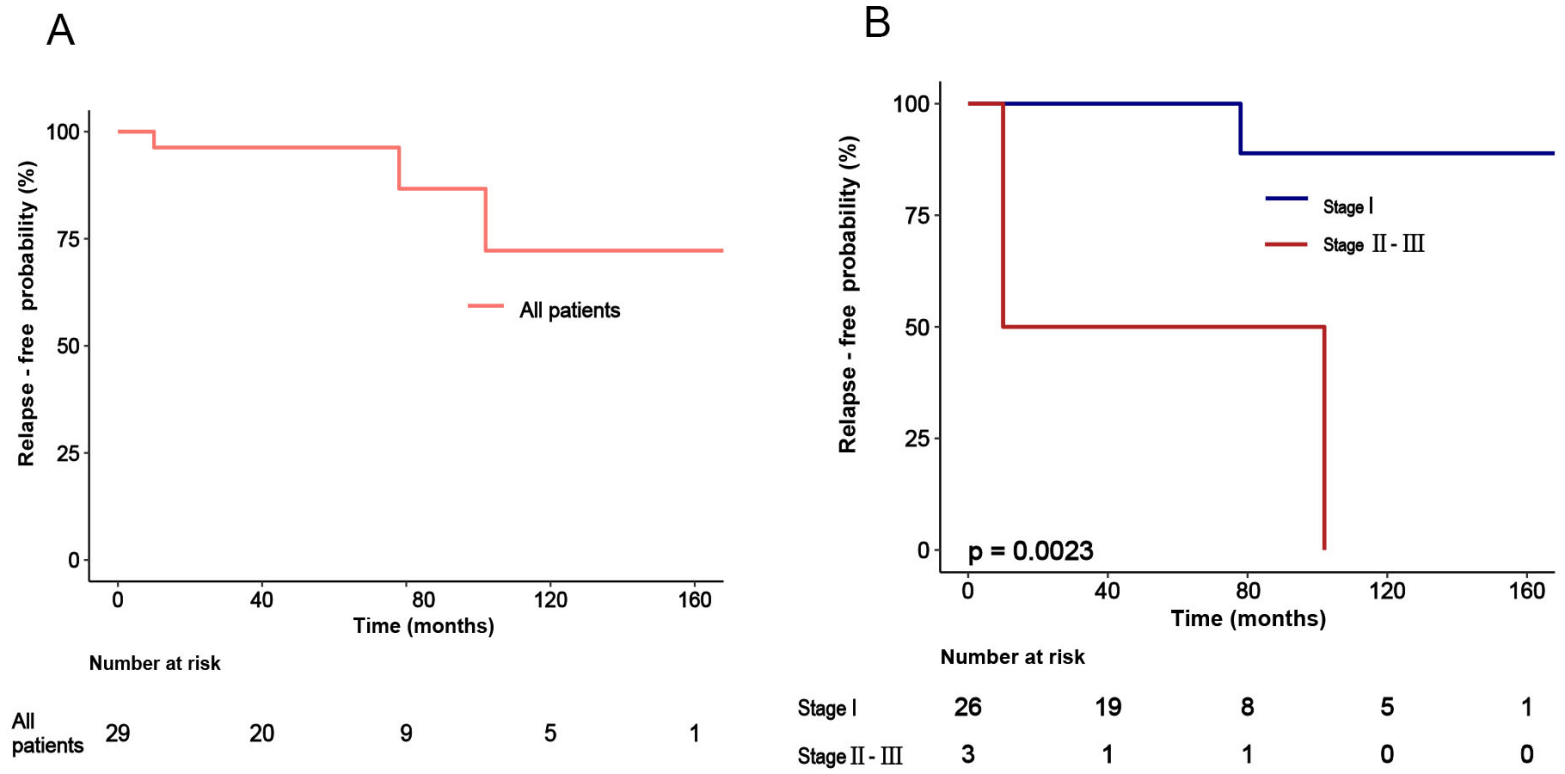
Relapse-free survival in ovarian granulosa cell tumors. **(A)** Overall relapse-free survival for the entire cohort (n=29), including 26 adult-type and 3 juvenile-type cases. Three recurrence events occurred during the observation period, all in adult-type cases (median follow-up 52.5 months, IQR 19.5-82.5). No recurrences occurred in the three juvenile-type cases (follow-up: 76, 96, 124 months). **(B)** Relapse-free survival stratified by FIGO stage in granulosa cell tumors (n=29). Advanced stage disease (II-III) was associated with significantly worse RFS compared to stage I (log-rank P = 0.0023).

Kaplan-Meier analysis showed that patients with FIGO stage II-III had significantly worse relapse-free survival (RFS) compared to those with stage I disease (p = 0.0023) (**Figure 2**).

### Prognostic factor analysis

Given the small juvenile cohort (n=3) with zero events during follow-up, prognostic factor analysis using Cox regression was restricted to adult-type GCTs (n=26, 3 events). Univariate Cox regression analysis identified FIGO stage as a significant prognostic factor for RFS. Patients with FIGO stage II-III tumors demonstrated a significantly higher recurrence risk compared to those with stage I disease (HR: 13.9, 95% CI: 1.21-160.24, p = 0.035). CA125 levels showed a trend toward significance (HR: 1.04, 95% CI: 1.01-1.08, p = 0.054). Adjuvant chemotherapy showed a numerical trend (HR 5.34, P = 0.182), likely reflecting confounding by indication as chemotherapy was administered to higher-risk patients.

In multivariate analysis including FIGO stage, CA125, and adjuvant chemotherapy (variables with P<0.20), advanced FIGO stage remained the only independent prognostic factor (HR 13.57, 95% CI 1.19-154.3, P = 0.035). CA125 showed a trend (HR 1.03, 95% CI 1.00-1.06, P = 0.079) that did not reach statistical significance after adjustment. The wide confidence intervals reflect the limited number of events (n=3) available for analysis.

Other factors, including age, tumor size, chemotherapy administration,surgical staging, and tumor markers (ROMA1, ROMA2, AFP, CEA, HE4), did not demonstrate significant associations with RFS in either univariate or multivariate analyses (**Table 5**).

**Table 5:** Prognostic factors for relapse-free survival in adult-type granulosa cell tumors.

Factors	Univariate analysis RFS		Multivariate analysis RFS	
	HR (95% CI)	p value	HR (95% CI)	p value
Age, years (≥ 45 vs. < 45)	0.3 (0.02 - 4.76)	0.39		
FIGO Stage (II-III vs. I)	13.9 (1.21 -160.24)	0.035	13.57 (1.19 -154.25)	0.035
Tumor Size (≤10cm vs. >10cm)	0 (0 - Inf)	0.999		
Surgical Staging (incomplete vs. complete	1.57(0.14 - 17.46)	0.714		
Adjuvant Chemotherapy (No vs. Yes)	5.34 (0.46 - 61.34)	0.182	7.27(0.49 - 107.77)	0.149
ROMA1	1.02 (0.90 -1.14)	0.887		
ROMA2	1.02(0.90 -1.15)	0.884		
AFP	0.64 (0.17 - 2.46)	0.515		
CEA	0.68 (0.15 - 3.24)	0.604		
CD125	1.04 (1.00 - 1.08)	0.054	1.03 (1.00 -1.06)	0.079
HE4	1.01(0.99 - 1.05)	0.684		

Analysis restricted to adult-type granulosa cell tumors (n=26) with 3 recurrence events during median follow-up of 52.5 months (range 9–168 months.Juvenile-type cases (n=3) were excluded from prognostic analysis due to zero recurrence events during the follow-up period (median 96 months, range 74–124 months) and insufficient sample size.

### Juvenile-type outcomes

All three juvenile-type cases (ages 17, 22, 28 years) presented with early-stage disease (stage I), underwent fertility-sparing surgery, and remained disease-free during follow-up of 76, 96, and 124 months. However, the small sample size and zero events preclude formal survival analysis or meaningful conclusions about long-term prognosis in this subgroup.

## Discussion

This retrospective cohort analysis provides a comprehensive understanding of the clinical features and outcome predictors of OGCTs, specifically highlighting the differences between adult-type and juvenile-type variants. Our findings contribute valuable insights to the limited literature on these rare tumors and have important implications for clinical management and patient counseling.

The predominance of adult-type GCTs (89.7%) in our cohort aligns with previous reports (19, 20), as these tumors typically affect postmenopausal women, with a peak incidence in the fifth to sixth decades of life (21). The significant age difference observed between adult-type (median 50.5 years) and juvenile-type (median 13.0 years) tumors reflects established epidemiological patterns (22, 23), emphasizing age as a key distinguishing factor between these subtypes.

The presenting symptoms in our study, with abdominal mass being the most common manifestation (51.7%), reflect the indolent nature of GCTs (24). Unlike epithelial ovarian cancers, which often present with advanced disease, the majority of our patients (89.7%) presented with FIGO stage I disease, consistent with the favorable prognosis typically associated with early-stage GCTs (25, 26). This finding highlights the importance of maintaining a high clinical suspicion for GCTs in patients presenting with pelvic masses, particularly in those with hormonal symptoms or abnormal bleeding patterns (27).

Tumor marker analysis reveals patterns that warrant clinical attention. While CA125 elevation was observed in 48.3% of patients, the median level of 33.7 U/mL is considerably lower than that typically seen in epithelial ovarian cancer. This, combined with the trend toward prognostic significance (p = 0.076), suggests that CA125 may serve a different role in GCTs—likely reflecting tumor burden rather than malignant transformation (28). The HE4 and ROMA indices showed numerically higher values in juvenile-type tumors, although statistical significance was not reached, likely due to the small sample size of this subgroup (15). These findings indicate that while traditional ovarian cancer markers may be elevated in GCTs, their interpretation should be approached with caution and in conjunction with clinical and imaging data.

The immunohistochemical profile observed in our study reinforces the reliability of established markers for diagnosing GCTs. High positivity rates for inhibin-α (93.1%), calretinin (92.3%), and CD99 (95.0%) are consistent with previous reports (29, 30) and support their continued use in diagnostic algorithms. The uniform expression of these markers across both adult-type and juvenile-type tumors suggests that immunohistochemical differentiation between subtypes primarily relies on morphological features rather than marker expression patterns.

The Ki-67 proliferation index findings, with elevated levels (> 30%) observed exclusively in adult-type tumors, warrant particular attention. While previous studies have suggested that high Ki-67 levels may correlate with more aggressive behavior (30), the limited number of recurrence events in our cohort prevents definitive conclusions about its prognostic significance. Larger studies are needed to more comprehensively investigate the relationship between proliferation markers and clinical outcomes.

The significant difference in surgical management between adult-type and juvenile-type tumors (p = 0.018) reflects clinical decision-making based on patient age and reproductive desires. The universal use of fertility-sparing surgery in juvenile-type cases highlights the importance of preserving reproductive function in young patients, especially considering the generally favorable prognosis of early-stage disease. This approach is supported by emerging evidence suggesting that fertility-sparing surgery does not compromise oncological outcomes in carefully selected patients with stage I GCTs (31, 32).

The marked contrast in surgical approaches between adult-type (76.9% radical surgery) and juvenile-type tumors (100% fertility-sparing) appropriately reflects patient demographics and reproductive priorities. Our findings align with emerging evidence that fertility-sparing surgery does not compromise oncological outcomes in early-stage disease (11). However, the 19.2% rate of fertility-sparing surgery in adult-type cases suggests that conservative management could be expanded for younger patients. Given the excellent prognosis of stage IA disease and advances in assisted reproductive technologies, criteria for fertility preservation may need to be relaxed, particularly for women under 40 with unilateral, encapsulated tumors.

Our identification of FIGO stage as an independent prognostic factor (HR: 13.93, p = 0.034) reinforces the critical importance of accurate staging in GCTs. This finding is consistent with multiple previous studies (25, 33, 34) and supports the continued use of the FIGO staging system for risk stratification and treatment planning. The substantial hazard ratio suggests that patients with advanced-stage disease require more intensive surveillance and consideration of adjuvant therapy.

The borderline significance of CA125 as a prognostic factor (p = 0.076) is intriguing and warrants further investigation in larger cohorts. Although CA125 is not traditionally considered a primary prognostic marker for GCTs, our findings suggest it may have utility in risk assessment and monitoring. This observation supports the inclusion of serial CA125 measurements in long-term surveillance protocols.

Our findings support several clinical recommendations. First, the favorable outcomes associated with early-stage disease emphasize the importance of timely diagnosis and appropriate surgical management. Second, the feasibility of fertility-sparing surgery should be discussed with all premenopausal patients in selected cases. Third, long-term surveillance is critical due to the potential for late recurrence, with CA125 monitoring recommended alongside clinical assessment.

Given the rarity of GCTs, collaborative efforts are needed to advance understanding. Key areas for future investigation include: (1) molecular profiling to identify novel prognostic markers beyond traditional clinicopathological factors; (2) standardization of adjuvant therapy indications for high-risk patients; (3) development of risk-adapted surveillance protocols incorporating modern biomarkers; and (4) evaluation of targeted therapies that exploit the unique biology of GCTs, particularly their hormonal responsiveness.

## Limitations

Several limitations should be acknowledged. First, the retrospective, single-institution design and small sample size (29 patients, with only 3 recurrences and 3 juvenile-type tumors) limit generalizability and statistical power. With only 3 events, our multivariate Cox regression analysis is underpowered (the traditional EPV rule suggests 10 events per variable), resulting in wide confidence intervals (HR 13.93, 95% CI 1.22-159.01), indicating model instability. These findings should be regarded as hypothesis-generating rather than definitive.

Second,the absence of GCT-specific tumor markers represents a significant limitation. Inhibin B and AMH, which demonstrate superior sensitivity (67-89%) and specificity (>90%) compared to CA125, were not routinely measured at our institution during the study period (2008-2024). Serum inhibin A was available for only 6 patients (20.7%), and preoperative estradiol for only 4 patients (13.8%), precluding meaningful analysis. Our reliance on non-specific markers like CA125 (elevated in only 40-60% of GCTs) may have compromised early recurrence detection. Future prospective studies should mandate GCT-specific marker panels (Inhibin B, AMH) and molecular profiling (FOXL2, TERT mutations) for improved risk stratification and disease monitoring.

Third, the absence of standardized imaging protocols and the median follow-up of 60 months (75% of patients < 8 years), while adequate for many malignancies, may be insufficient to capture the full spectrum of late recurrences typical of GCTs, which can occur decades after initial treatment.

## Conclusions

Within the constraints of our limited follow-up duration and modest sample size, this study identifies FIGO stage as the primary prognostic determinant in adult-type ovarian granulosa cell tumors. Early-stage disease demonstrates favorable short- to intermediate-term outcomes with appropriate surgical management. Fertility-sparing surgery appears feasible in early-stage disease, particularly for juvenile-type tumors, though longer follow-up is needed to confirm oncologic safety.

Our reliance on CA125 reflects institutional practice constraints but represents a significant limitation. The borderline CA125 significance (P = 0.050 univariate, P = 0.079 multivariate) should not validate its use; this marginal association likely reflects tumor burden correlation rather than GCT-specific utility. Contemporary evidence supports Inhibin B and AMH as superior markers, and future studies must incorporate these GCT-specific biomarkers along with molecular profiling (FOXL2, TERT) for improved risk stratification.

Future multicenter collaborations are essential to achieve adequate sample sizes and extended follow-up (minimum 10–15 years for adult-type GCTs). Key priorities include establishing standardized surveillance protocols with GCT-specific biomarkers, developing risk-adapted treatment algorithms balancing oncologic safety with fertility preservation, and molecular profiling to understand distinct tumor biology. While our study’s limitations preclude definitive conclusions, it provides hypothesis-generating data highlighting critical needs for future research in this rare malignancy.

## Data Availability

The raw data supporting the conclusions of this article will be made available by the authors, without undue reservation.
